# Advances in plasma metabolomics detection technology and its clinical applications in lung cancer and other malignancies

**DOI:** 10.1007/s44178-026-00248-x

**Published:** 2026-04-24

**Authors:** Yue He, Juntong Guo, Pufan Gao, Zifan Li, Wenxiang Wang, Kezhong Chen

**Affiliations:** 1 Department of Thoracic Surgery, Peking University People’s Hospital, 11 Xizhimen South Street, Beijing, 100044 China; 2 Peking University People’s Hospital Thoracic Oncology Institute, Beijing, 100044 China; 3 Research Unit of Intelligence Diagnosis and Treatment in Early Non-Small Cell Lung Cancer, Chinese Academy of Medical Sciences, 2021RU002, Peking University People’s Hospital, Beijing, 100044 China; 4 Peking University Health Science Center, Beijing, 100191 China

**Keywords:** NSCLC, Cancer metabolism, Clinical application, Liquid biopsy

## Abstract

Metabolic reprogramming is a fundamental hallmark distinguishing tumor cells from their normal counterparts. This process leads to pronounced alterations in the types and concentrations of metabolites present in the bodily fluids of cancer patients via liquid biopsy approaches. Plasma and serum, owing to their minimally invasive collection, repeatability, and ability to reflect systemic metabolic status, have emerged as optimal sample types for clinical metabolomics. These metabolic changes serve as valuable indicators for inferring disease progression and predicting patient prognosis. Lung cancer, particularly non-small cell lung cancer (NSCLC), with its high global incidence and mortality, represents a critical area where plasma metabolomics can address unmet clinical needs in prognostic prediction and therapeutic stratification. This review focuses primarily on lung cancer. However, the scarcity of studies investigating the prognostic value of metabolite alterations in lung cancer promoted the inclusion of research from other malignancies as well. This review first summarizes the current liquid biopsy metabolomics detection technologies and associated biological materials, followed by an overview of tumor-related metabolic pathway alterations. It then discusses the clinical applications of these principles in prognostic prediction and therapeutic evaluation. The aim is to provide a comprehensive overview and inspire future research directions in this field.

## Introduction

Each year, over 10 million individuals are diagnosed with cancer worldwide, and nearly 8 million succumb to cancer or related diseases. Accounting for approximately 12% of global mortality, positioning cancer remains one of the leading causes of death. Lung cancer, in particular, ranks among the highest in both incidence and mortality. To facilitate precision medicine and enhance survival rates, accurate prognostic predictions are imperative. Currently, however, simple, reliable, and clinically applicable tools for prognostic prediction remain lacking. This review explores the prognostic potential of metabolites, summarizing the major liquid biopsy techniques for metabolite analysis in recent years, tumor-associated metabolic pathway alterations, and the correlations between specific metabolite signatures and prognosis, with a focus on non-small cell lung cancer (NSCLC).

Tumor cells undergo substantial changes in multiple metabolic pathways, including glucose, lactate, glutamine, and lipids metabolisms, leading to measurable shifts in metabolite levels. While metabolomics has advanced rapidly and been widely applied in early cancer screening, study directly linking metabolite levels to cancer prognosis remains comparatively limited.

Metabolic activities underpin essential physiological functions, and metabolites provide a dynamic representation of the functional state of tissues or individuals [1]. When cellular physiological functions are altered, such as following mutations that lead to tumorigenesis, metabolic processes frequently shift, resulting in changes in metabolite levels. Many tumors generate characteristic metabolite signatures or altered concentrations, which often correlate with disease progression and prognosis. Consequently, the detection of changes in characteristic metabolites represents a viable approach for predicting tumor prognosis. Common biospecimens used for metabolomics include plasma, serum, urine, tissue samples, along with other biological materials such as sweat, exhaled breath condensate (EBC), bronchoalveolar lavage fluid (BALF), cerebrospinal fluid (CSF), and sputum. Plasma and serum remain the most frequently used due to their ability to reflect the systemic metabolism [2] and the advantages of liquid biopsy, including minimal invasiveness, ease of repeated sampling, and the ability to capture features of both primary and metastatic tumors [3, 4].

Because metabolic patterns vary across tumor subtypes and disease stages [5], prognostic analyses based on metabolites require careful consideration of these factors. For instance, metabolites such as carnitine, amino acids, lipids, and fatty acids demonstrate significant differences between early and late-stage NSCLC patient samples, and pronounced alterations in N-acyl ethanolamine (NAE) biosynthesis pathways have been observed between adenocarcinoma and squamous cell lung carcinoma subtypes [6].

Liquid biopsy based metabolite detection has already been widely employed for early tumor screening with high accuracy [7]. However, its applications in prognostic prediction and treatment selection are still developing. Therefore, this review is organized to provide a comprehensive understanding of the translational pathway from technology to clinical application: We first describe the current liquid biopsy technologies available for metabolite detection (Part 2), establishing the technical foundation for metabolomic analysis. Subsequently, we examine the specific metabolic pathway alterations that occur in tumors (Part 3), explaining which metabolites can serve as biomarkers. Finally, we synthesize these findings by presenting clinical applications (Part 4), demonstrating how technological capabilities and metabolic insights translate into prognostic and therapeutic tools. This integrated approach aims to bridge the gap between basic metabolic research and clinical oncology practice.

## Technology

Circulating tumor cells (CTCs), circulating tumor DNA (ctDNA), non-coding RNA, extracellular vesicles (EVs), tumor metabolites, tumor-associated antigens, and tumor-educated platelets are often considered as promising biomarkers for liquid biopsy of cancer. This review focuses on liquid biopsy of metabolites.

### Common technologies

**Fig. 1:**
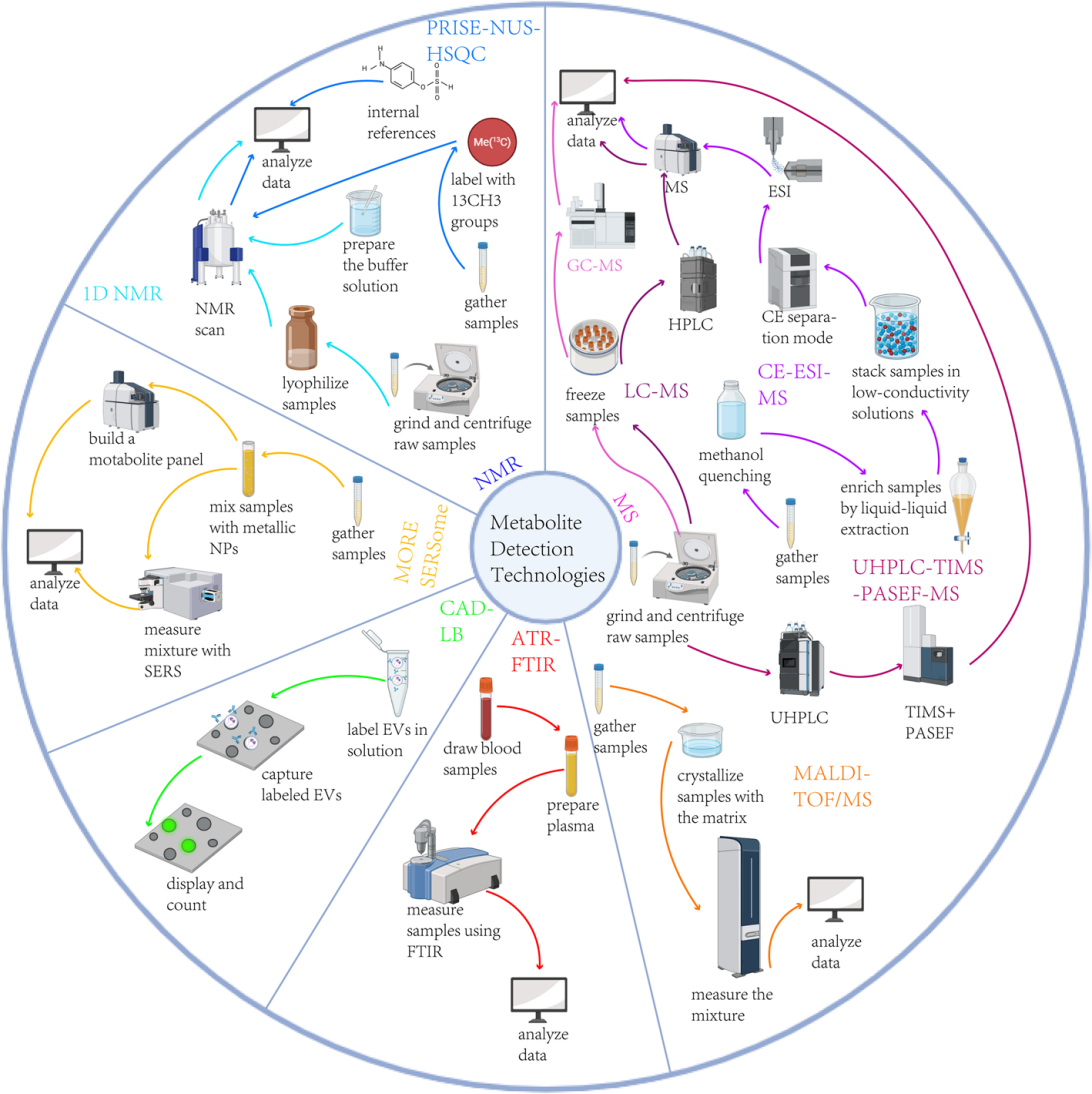
Metabolite detection techniques This figure presents various common techniques for metabolite detection, as well as the latest methodologies discussed. The arrows, differentiated by color, denote the respective techniques. NMR, nuclear magnetic resonance; 1D NMR, 1 dimensional nuclear magnetic resonance; PRICE-NUS-HSQC, probe-induced sensitivity enhancement and nonuniform-sampling-based 1H-13C heteronuclear single quantum coherence 2 dimensional nuclear magnetic resonance; MS, mass spectrometry; GC-MS, gas chromatography-mass spectrometry; LC-MS, liquid chromatography-mass spectrometry; HPLC, hyphenated to liquid chromatography; CE-ESI-MS, capillary electrophoresis-electrospray ionization-mass spectrometry; UHPLC, ultra-high-performance liquid chromatography; TIMS, trapped ion mobility spectrometry; PASEF, parallel accumulation serial fragmentation; MALDI-TOF/MS, matrix-assisted-laser desorption/ionization time of flight mass spectrometry; MORE SERSsome, molecule-resolvable surface-enhanced Raman spectroscopy; NP, nanoparticle; CAD-LB, catch and display for liquid biopsy; EVs, extracellular vesicles; ATR-FTIR, attenuated total reflectance Fourier-transform infrared spectroscopy

The main analytical methods employed in metabolomics are nuclear magnetic resonance (NMR) and mass spectrometry (MS) technologies (Fig. 1). NMR technology necessitates minimal sample preparation [8] and enables rapid, high-throughput, quantitative, and reproducible measurement of metabolites [9]. However, it is characterized by relatively lower sensitivity and comparatively higher costs (Table 1).

**Table 1:** Summary of Common Metabolite Detection Techniques

Common technologies	Common samples	Analysis time	Sample volume	LOD	Approximate cost per sample	Throughput	Advantages	Disadvantages
NMR	Tissue, Body fluid, Urine	2–3 min [10]	0.1–0.5 mL [10]	5 μM [10]	$50–$150 [10]	72 plasma/serum samples per 24 h [11]	Fewer sample preparation steps; High-throughput; Quantitative; Reproducible [12–14]	Low sensitivity; High cost [10]
GC–MS	Tissue, Urine, Body fluid, Exhaled breath condensate, Bronchoalveolar lavage fluid	20–40 min [10]	0.1–0.2 mL [10]	0.5 μM [10]	$20–$60 [10]	47 samples per 24 h [12]	More suitable for small molecules; High accuracy [15–17]	Low detection capacity; Limited range of detectable substances [10]
LC–MS	Tissue, Body Fluid, Sweat, Urine, Saliva	15–40 min [10]	10–100 μL [10]	0.5 nM [10]	$30–$100 [10]	120 samples per 4.5 days [12]	High universality; Capable of detecting a wide variety of metabolites; Be suitable for non-targeted metabolomics [16–19]	Be susceptible to the effects of batch variation and background interference [10]
CE-MS	Serum, Plasma, Urine, Saliva, Cerebrospinal Fluid, Bile, Amniotic fluid, Tears, Pancreatic juice, Intestinal fluid, Breast milk	30–60 s	20nL [20]	2.9–83.1 nM [21]	$10–$40	-	Low required sample size; Fast separation speed	Worse system stability than that of LC–MS and GC–MS; Affected by the salt content in the samples [22]; Inferior concentration sensitivity [21]
HPLC	Blood, Plasma, Urine	10–30 min	10–20μL	0.3 ng/mL	$10–$50	120 samples per 4.5 days [12]	High separation efficiency; High detection sensitivity; Fast speed [23]	Poor long-term stability; Limited resolution for complex samples; Complicated operation
MALDI-TOF/MS	Serum, Urine, Tears, Cerebrospinal fluid	a few seconds—2 h [24]	Approximately 600μL [24]	0.2 pmol [25]	$5–$30	Sample run time seconds to minutes	Streamlined sample preparation process; Elevated sensitivity, Robust tolerance to saline conditions; Swift operation; High sample processing capacity	Poor reproducibility; Poor multifunctionality; Prone to ion source contamination [26]
ART-FTIR	Blood, Urine [27]	Approximately 30 s	Approximately 6μL [28]	10–30 mg/mL [29]	Low operational cost; major cost in instrument (~ $50,000–100,000 range)	~ 30 s per sample	High chemical specificity, High spatial resolution, Large amount of information [30]	Limited yield and stability of the grafted molecules on germanium [31]
CAD-LB	Blood, Urine, Saliva, Cerebrospinal fluid, Breast milk, Amniotic fluid, Semen, Bile	-	Approximately 40μL [32]	-	-	-	High throughput; Reproducibility; Capable of achieving unbiased quantification and assessment	Narrow scope of application for exosome detection
FT-ICR-MS	Serum, Pasma	Approximately 5 min	0.5μL	-	Very high capital cost (> $1 million); operational cost estimated **$100 + **per sample	12 samples per day (5 min per analysis)	Extremely high resolution and sensitivity; Low mass error	Only applicable to small molecules [33]
TIMS + PASEF	Cell culture, Single cells Tissues, Body fluids	Approximately 35 min	5–200 ng [34]	-	$50–$150	20–30 samples per day	High ion utilization; High scan rate; High sensitivity to low-abundance substances [35]	Limited ability to handle highly complex samples
MORE SERSome [36]	Serum, Urine, Cell	Less than 4 min	10μL [37, 38]	-	$10–$30	< 4 min per sample	Molecule-level resolution	Neglect some molecular species; Low ionization efficiency; Poor NP affinity

MS-based metabolomics offers high sensitivity, specificity, and robust data acquisition. Traditional MS technologies mainly include capillary electrophoresis-mass spectrometry (CE-MS), gas chromatography-mass spectrometry (GC–MS), and liquid chromatography-mass spectrometry (LC–MS). Among these, CE-MS is a powerful analytical separation technique particularly effective for the efficient analysis of polar and charged metabolites, including classes such as amino acids, nucleotides, small organic acids, and phospho-sugars. Historically, CE-MS was perceived to exhibit lower reproducibility compared to GC–MS and LC–MS; however, recent technological advancements have significantly enhanced its throughput, rendering this technique more practical [39]. Additionally, innovations such as the sheathless interface have improved the sensitivity of CE-MS [40]. GC–MS is widely employed for the assessment of small molecules but but has limitations in high-throughput and unidentified compound detection. In contrast, metabolic profiling based on LC–MS demonstrates high sensitivity and good universality, allowing for the specific identification of various metabolites at low concentrations. This capability has established LC–MS as the preferred method for untargeted metabolomics [41–44]. Traditional chromatographic techniques, including high-performance liquid chromatography (HPLC) and gas chromatography (GC), also play important roles. GC necessitates the conversion of substances into a gaseous state, rendering it suitable only for small molecular compounds that are stable at elevated temperatures, while HPLC offers a broader range of applications. Despite the widespread application of these methods in metabolomic analysis, traditional approaches still encounter technical barriers, such as difficulties in preserving spatial information and ensuring enhanced sensitivity across diverse application contexts [45].

In recent years, advancements and refinements in metabolomic detection technologies have facilitated the widespread application of these techniques in clinical settings. Advancements in metabolomics technologies have introduced novel metabolite detection techniques. The Catch and Display for Lipid Biopsy (CAD-LB) specifically targets the detection of EVs, allowing for their separation and the counting of biomarkers, thus serving as a "digital" detection method [46]. High-resolution mass spectrometry (HRMS) represents the latest evolution in mass spectrometry. HRMS, particularly Fourier transform ion cyclotron resonance (FT-ICR-MS), offers exceptional resolution and sensitivity for precise mass determination. To better exploit low-abundance ions in complex samples, the parallel accumulation–serial fragmentation (PASEF) approach, integrated with trapped ion mobility spectrometry (TIMS), markedly improves peak capacity and identification depth [47]. Probe-induced sensitivity enhancement and nonuniform-sampling-based ^1^H-^13^C heteronuclear single quantum coherence 2D-NMR(PRISE-NUS-HSQC) leverages probe sensitization to break through the micromolar detection limit of NMR, drastically improving its sensitivity [48]. Furthermore, the molecule-resolvable surface-enhanced Raman spectroscopy (MORE SERSome) technique combines laser desorption/ionization mass spectrometry with SERS to identify dominant metabolic molecules in serum, enabling specific molecular resolution and relative quantification of label-free SERS spectra [36]. These technological advancements have significantly enhanced the sensitivity and accuracy of metabolite detection techniques, laying a solid technical foundation for the clinical application of metabolite detection.

### Common samples

A review of recent lung cancer studies found that in NMR, tissue samples account for ~ 31% and plasma/serum for ~ 54%; in GC–MS, tissue represents ~ 5% and plasma/serum ~ 62%; and in LC–MS, tissue also accounts for ~ 5% and plasma/serum ~ 65% [1]. These findings indicate that liquid biopsy-based metabolite detection is employed more frequently than tissue-based metabolite profiling [49]. This preference may result from its repeatable and non-invasive nature, which facilitates sampling and minimizes patient harm [50, 51]. Moreover, due to tumor heterogeneity, tissue biopsy may fail to capture the complete mutational landscape, whereas liquid biopsy can partly overcome this limitation.

Nevertheless, several constraints of liquid biopsy should be acknowledged. Current technologies still suffer from limited detection accuracy [52], and metabolites in liquid samples exhibit instability and insufficiency in reflecting the body’s status. Complex samples such as plasma are susceptible to minor metabolic fluctuations in specific body regions; samples possess poor stability, with certain components undergoing rapid degradation and transformation once after sample collection; and the intricate composition of metabolites makes it difficult to comprehensively measure the metabolome in a single analysis.

Following sample collection, different pretreatment procedures are required depending on the detection technology employed. NMR imposes relatively minimal requirements, often requiring no pretreatment or separation, or only simple centrifugation and fractionation. In contrast, conventional MS demands high sample purity, necessitating the removal of high-molecular-weight compositions through centrifugation or other purification steps. For spatial metabolomics, MS-based technologies typically require tissue preservation through freezing or paraffin embedding [53]. Numerous factors during these pre-analytical phases pose threats to sample stability. For example, delayed centrifugation of whole blood significantly alters levels of glucose, potassium, and aspartate aminotransferase [54]; non-refrigerated storage leads to substantial degradation of specific components in serum or plasma within a short period [55, 56]; and excessive freeze–thaw cycles can markedly change biomarker profiles [57]. The complex and dynamic alteration of metabolites poses challenges to its application. Such challenges underscore the importance of strict sample processing protocols to ensure accuracy and reproducibility of metabolomics detection [49, 58].

To address the limitations imposed by unavoidable metabolite instability, researchers have also attempted to integrate metabolomics with other omics, expecting multi-omics to compensate for inaccuracies in single metabolomics and further validate discovered metabolic pathways. The table below (Table 2) lists several studies that apply multi-omics(including metabolomics) to investigate the molecular metabolic mechanisms of cancers, especially that of lung cancer.

**Table 2:** Studies that integrated metabolomics with other omics to profile the molecular mechanisms of cancer

-omics	Cancer type	Purpose(s)	Advancements	Reference
Metabolomics, Microbiota metagenomics	Non-small cell lung cancer	Treatment	Higher reliability, More personalized therapeutic targets	[59]
Metabolomics, Global lactylomics	Non-small cell lung cancer	Treatment	Clearer pathway, More effective therapeutic targets	[60]
Metabolomics, Genomics, Transcriptomics, Proteomics	Lung cancer brain metastases	Treatment	Clearer pathway, More precise medicine	[61]
Metabolomics, Genoomic, Proteomics	Glioblastoma	Stratification	Clearer molecular pathogenesis	[62]
Metabolomics, Genomics, Transcriptomics, Proteomics, Radiomics	Breast cancer	Stratification, Treatment	More personalized therapeutic targets	[63]
Metabolomics, Microbiota metagenomics	Colorectal cancer	Diagnosis	More specific biomarkers	[64]

Although relative few reports exist thus far, we believe there is strong potential for integrating metabolomics with other omics for clinical application in lung cancer and other cancer-related fields. Notably, at the 2023 World Conference on Lung Cancer (WCLC), a "classifier" based on metabolomics, proteomics, and transcriptomics profiling of peripheral blood was introduced, demonstrating excellent performance in both early-stage and full-stage lung cancer detection [65] and showing promise for future clinical use.

## Altered metabolic pathways in tumors

**Fig. 2:**
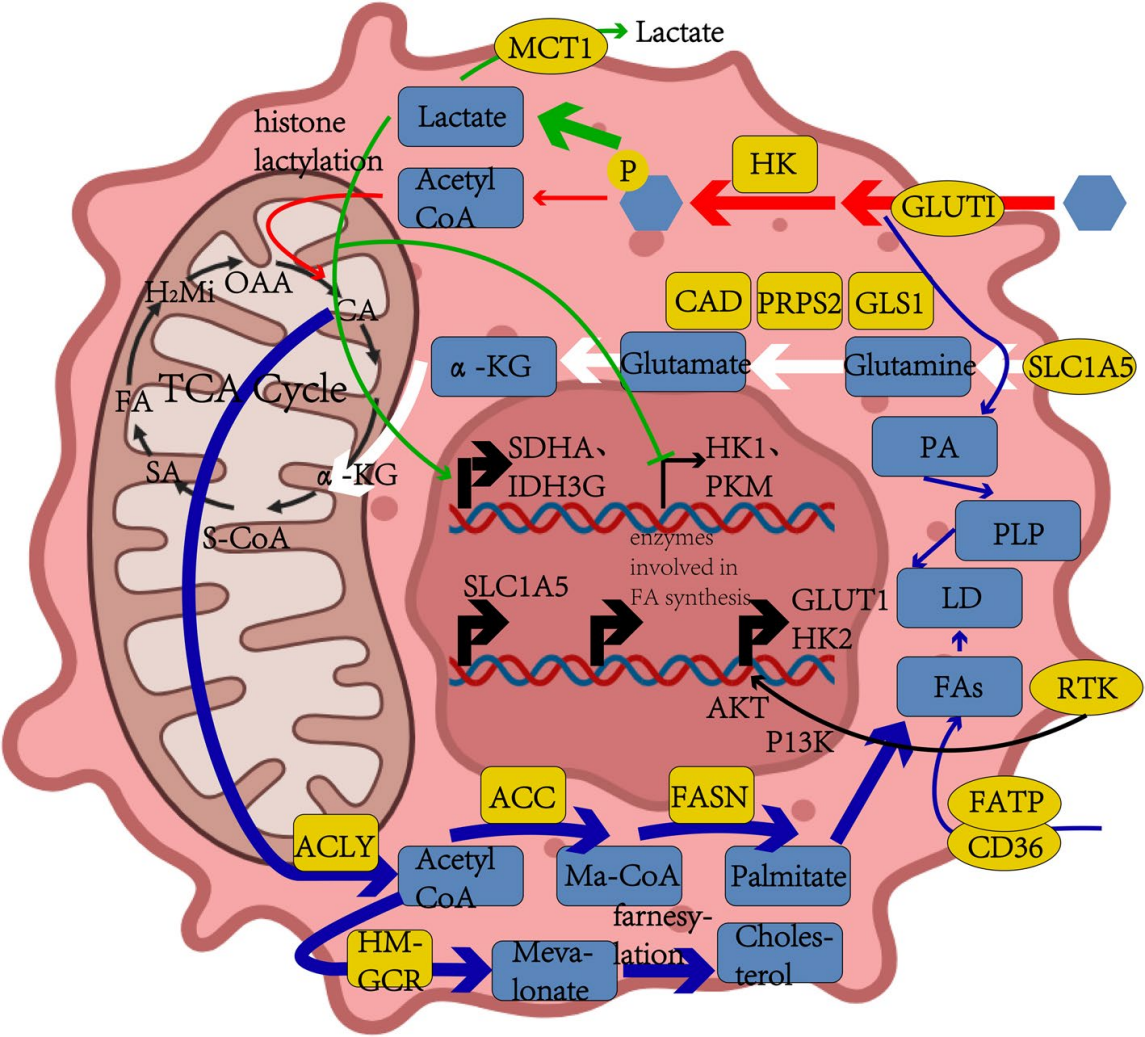
Altered metabolic pathways in tumor This figure illustrates four key metabolic pathways altered in tumor tissues, with arrow thickness indicating the relative flux changes (increased or decreased activity). CA, citrate acid; α-KG, α-ketoglutarate; S-CoA, succinyl-coenzyme A; SA, succinate acid; FA, fumarate acid; H2Mi, malic acid; OAA, oxaloacetic acid; GLUT1, glucose transporter 1; HK, hexokinase; MCT1, monocarboxylate transporter 1; SLC1A5, solute carrier family1, member5; PA, phosphatidic acid; PLP, phospholipid; LD, lipid droplets; FAs, fatty acids; FATP, fatty acid transport proteins; CD36, cluster of differentiation 36; ACLY, acetyl-coenzyme A by ATP-citrate lyase; ACC, acetyl-coenzyme A carboxylase; FASN, fatty acid synthase; HM-GCR, 3-hydroxy-3-methylglutaryl coenzyme A reductase; Ma-CoA, malonyl-coenzyme A; AKT, protein kinase B; PI3K, phosphatidylinositol 3-kinase; RTK, receptor tyrosine kinase; CAD, carbamoyl-phosphate synthetase 2, aspartate transcarbamylase and dihydroorotase; PRPS2, phosphoribosyl pyrophosphate synthase 2; GLS1, glutaminase 1; SDHA, succinate dehydrogenase; IDH3G, isocitrate Dehydrogenase (NAD(+)) 3 Non-Catalytic Subunit Gamma; PKM, pyruvate kinase M

Cancer cells and cancer-associated fibroblasts (CAFs) display profound alterations in gene expression relative to normal cells [66]. Dysregulation of specific genes directly influences glucose uptake and utilization, thereby driving global metabolic reprogramming in cancer cells [67]. The key metabolic hallmarks of tumors include (Fig. 2) [68]: aberrant uptake of glucose and amino acids [69] [70]; diversification of nutrient acquisition pathways; preferential reliance on glycolysis and tricarboxylic acid (TCA) cycle intermediates (the Warburg effect); elevated nitrogen demand; metabolite-mediated dysregulation of gene expression; and dynamic interactions between metabolites and the tumor microenvironment [71].

Glucose uptake in tumor cells is mediated primarily by glucose transporter 1 (GLUT1) and its downstream enzyme hexokinase (HK), under the regulation of the phosphatidylinositol 3-kinase–protein kinase B (PI3K-AKT) signaling pathway [72]. The large neutral amino acid transporter (LAT1) facilitates the import of substrate amino acids for glutamine synthesis, a process transcriptionally regulated by the oncogene c-MYC [73]. During active proliferation, tumor cells preferentially engage in anaerobic glycolysis, producing lactate and generating intermediates such as nicotinamide adenine dinucleotide phosphate (NADPH) to support biosynthetic processes, rather than directing glycolytic products into the TCA cycle. In this context, glutamine serves as the primary substrate fueling the TCA cycle. To meet the elevated demand for nitrogen-containing metabolites during proliferation, tumor cells further enhance nitrogen acquisition through pathways such as arginase-1–ornithine decarboxylase (ARG1-ODC) (Table 3).

**Table 3:** Clinical trials on the association between metabolites and prognosis

Relevant Experiment/Article/Trail Number	Metabolic Pathway	Experimental Protocol	Pathway Changes	Metabolite Type	Metabolite Level	Prognosis/Therapeutic Effect
PMC9198346	Lipid	High-resolution MS analysis of surgical resection tissue samples of NSCLC obtained after neoadjuvant chemotherapy (NAC). [74]	Glycerophospholipid metabolism	Sphingomyelin (SM)	High	Good
				Lysophosphatidylinositol (LysoPI)	Low	Good
				Phosphatidylethanolamine (PE)	Low	Good
				Lysophosphatidylcholine (15:0/0:0)/Lysophosphatidylethanolamine (18:0/0:0)	High	Good
NCT01835041	Glucose and glutamine	Measurement of plasma metabolite levels in melanoma patients by NMR spectroscopy [75]	Increased PDHA1 expression, enhanced TCA cycle	Pyruvate	High	Bad
				Fumarate	High	Bad
				2-Oxoglutarate	High	Bad
NCT03504423	Glucose and glutamine	Folfirinox (FFX) combined with CPI-613 and modified Folfirinox (mFFX) for the treatment of pancreatic metastatic adenocarcinoma	Low expression of mitochondrial pyruvate carrier MPC1 and MPC2 [75, 76]	Pyruvate	High	Bad
NCT03089606	Tryptophan (Trp)	Subjects underwent C11-AMT PET, FDG PET, baseline study tumor biopsy, and received at least one infusion of pembrolizumab as part of the study	Disordered Trp metabolism [77]	Tryptophan	High	Bad
GSE115978, GSE123813	Lactate	Collection and review of publicly available single-cell RNA sequencing (scRNA-seq) cohorts obtained from patients receiving immunotherapy to elucidate the association between liver metastasis (LM) and immunotherapy response	Disordered lactate metabolism [78, 79]	Lactate	High	Bad
PMC11800462	Lactate, Leucine	The study included 33 healthy controls (HC), 38 newly diagnosed multiple myeloma patients (NDMM) divided into three RISS grades, and 92 multiple myeloma patients treated with bortezomib regimen after targeted therapy	Upregulated CKS2 expression, downregulated LYZ expression [80]	Lactate	Low	Bad
				Leucine	Low	Bad

Based on our discussion of detection technologies in Part 2, we propose that variations in the types and levels of plasma metabolites arising from tumor-driven metabolic pathway alterations can be systematically detected through modern metabolomics platforms. These measurable changes may then be leveraged for prognostic prediction, stratification, and early tumor screening.

### Altered glucose metabolic pathways in tumors

A hallmark of tumor metabolism is the increased glucose uptake, accompanied by a predominant reliance on aerobic glycolysis—known as the Warburg effect—for energy production, even in the presence of sufficient oxygen [81]. This stands in contrast to normal cells, which primarily utilize oxidative phosphorylation. This section explores these metabolic alterations from a genetic perspective, emphasizing key regulatory mechanisms. The emergence of the Warburg effect in tumor cells is primarily associated with several metabolic processes: glucose uptake, the transformation of glucose and its metabolites, and the eventual production of lactic acid, the end product of aerobic glycolysis. The following analysis will delve into the changes in metabolic pathways within tumor cells and the gene expression modifications that drive these alterations, with a particular focus on these processes.

Firstly, in the process of glucose uptake, glucose transporters such as GLUT1 and GLUT3 play a critical role. Mutations in the P53 and Akt genes can promote the translocation of GLUT1 to the plasma membrane [82–84]. Additionally, mutations in the hypoxia-inducible factor 1 alpha (HIF-1α) and Kirsten rat sarcoma viral oncogene homolog (Kras) genes can enhance the expression levels of the GLUT1 gene [85]. Furthermore, mutations in the HIF-1α gene can also upregulate the expression of the GLUT3 gene [82, 86–88]. In the transformation of glucose and its metabolites, the upregulation of various glycolytic enzymes facilitates aerobic glycolysis. HK catalyzes the initial step of glucose metabolism, and mutations in the HIF-1α and c-Myc genes enhance the expression of HK2 [82, 89, 90]. Phosphofructokinase (PFK) catalyzes the conversion of fructose-6-phosphate and ATP into fructose-1,6-bisphosphate and ADP during glycolysis. Mutations in the c-Myc, HIF-1α, and Kras genes result in increased expression levels of phosphofructokinase [82, 91–94]. Akt can activate the mechanistic target of rapamycin complex 1 (mTORC1). The PI3K/Akt/mTOR signaling pathway enhances glycolytic enzyme activity and promotes glycolysis by phosphorylating and activating key enzymes such as HK2 and 6-phosphofructo-2-kinase/fructose-2,6-bisphosphatase (PFKFB2) [82, 95]. Finally, in the process of lactate production, lactate dehydrogenase (LDH) plays a critical role. Mutations in the HIF-1α, c-Myc, and isocitrate dehydrogenase NAD(+)1/2 (IDH1/IDH2) genes lead to the overexpression of lactate dehydrogenase. Collectively, these factors contribute to the dysregulation of glucose metabolism in tumor cells.

High glycolysis not only provides sufficient energy for tumor cell growth but also facilitates tumor proliferation and invasion. For instance, studies investigating the role of glycolytic products in tumor growth have demonstrated that glycolysis generates significant amounts of lactic acid, which can lead to histone lactylation. This modification converts type I tumor-associated macrophages (TAMs), known for their anti-inflammatory properties, into type II TAMs. Type II TAMs, in turn, promote tumor growth and suppress anti-tumor immunity. Moreover, lactic acid can activate the nuclear factor kappa-light-chain-enhancer of activated B cells (NF-κB) in B cells, thereby increasing the expression of programmed death-ligand 1 (PD-L1) [96]. This further enhances the metabolic activity and immune suppression of macrophages [97, 98]. The glycolysis of cancer cells also has a significant impact on CD8 T cells and NK cells that exert anti-tumor immunity [99]. Tumor cells in the microenvironment consume glucose at a high intensity, creating an environment of glucose scarcity and a large amount of lactic acid. Glucose restriction weakens the anti-tumor effect of T cells [100]. Meanwhile, the research found that an excessively high lactic acid concentration in the microenvironment would damage the expression of activated T-cell nuclear factor (NFAT) in CD8 T cells and NK cells, ultimately reducing the production of IFN-γ [101, 102]. It is also important to note that metabolic pathways in CAFs will undergo corresponding changes, which may have an impact on the tumor immune environment. A study on breast cancer has shown that lactic acid produced by hypoxic CAFs provides fuel for metabolic coupling between CAFs and breast cancer cells [103]. By activating the TGF-β1/p38 MAPK/MMP2/9 signaling axis and supplying energy to the mitochondrial activity of cancer cells, it promotes the invasion of breast cancer cells.

Since the discovery of the Warburg effect, extensive research has explored glucose metabolic reprogramming in tumors. A large number of substances in this pathway demonstrate strong potentials for clinical application in evaluating tumor progression and predicting prognosis, which we will discuss in the "Clinical Application" section.

### Altered glutamine metabolic pathways in tumors

Compared to normal tissues, tumor tissues exhibit significantly higher rates of nutrient uptake and utilization. Glutamine metabolism in cancer cells shows distinct differences in synthesis, transport, and catabolic pathways when compared to normal cells. Below, we analyze how these metabolic disparities contribute to tumor growth and proliferation.

In ovarian cancer, CAFs produce elevated levels of glutamine through the action of glutamine synthetase (GS) [104]. Ovarian cancer cells import this glutamine, convert it to glutamate via glutaminase (GLS), and further metabolize glutamate to α-ketoglutarate (α-KG), which fuels the TCA cycle to support cell proliferation [105, 106]. Increased glutamine synthesis in the tumor microenvironment also enhances glycolytic activity and stimulates cell proliferation by activating the mTOR/S6 pathway [104, 107, 108].

Tumor cells exhibit altered expression of glutamine transporters, including SLC1A5, SLC6A14, SLC7A5, SLC7A11, and ASCT2 [109]. C-Myc binds to the promoter of SLC1A5, inducing its overexpression and enhancing glutamine influx [110]. SLC1A5 is critical for amino acid-induced mTORC1 activation, which amplifies metabolic reprogramming [111–113]. In estrogen receptor-positive breast cancer, SLC6A14 is upregulated and facilitates the uptake of neutral and basic amino acids [113–115]. Additionally, SLC7A5 mediates glutamine efflux in exchange for leucine, sustaining mTORC1 activation. Its overexpression accelerates cell proliferation by reducing G1-phase cell cycle arrest and activating AKT/mTORC1 signaling [111, 113, 116, 117]. The overexpression of SLC7A11 suppresses ferroptosis (driven by lipid peroxidation) to promote tumor progression [111, 113, 118–120]. In Osimertinib-resistant NSCLC cells, activation of the AMPK signaling pathway was observed [121]. This pathway enhances glutamine uptake by increasing the expression of ASCT2 [122].

Glutamine metabolism in tumors diverges through multiple pathways. Glutamine-derived ammonia (produced via GLS and GLUD) directly contributes nitrogen for purine and pyrimidine biosynthesis [111, 123]. Glutamine oxidation generates aspartate and α-KG, both precursors for nucleotide synthesis [124]. Under hypoxic conditions, the reductive carboxylation of glutamine produces citrate and fatty acids, while the synthesis of dihydroorotate helps mitigate ammonia toxicity [125, 126]. These metabolic adaptations collectively enhance nucleotide biosynthesis, maintain redox homeostasis, and promote biomass production, thereby driving unrestrained tumor growth and survival. Alterations in glutamine metabolism also have an impact on the function of immune cells. Typically, the predatory uptake of glutamine by tumor cells in the TME leads to limited utilization of glutamine by immune cells and affects the anti-tumor immune response. For instance, in glutamine-addicted clear cell renal cell carcinoma, the competitive uptake of glutamine by tumor cells leads to the deprivation of local extracellular glutamine. Glutamine activates HIF-1α and induces tumor-infiltrating macrophages to secrete IL-23. IL-23 further promotes the proliferation and activation of Treg cells, thereby inhibiting the anti-tumor activity of Teff cells [127]. Glutamine is crucial for the survival of B cells in hypoxic environments [128]. It simultaneously promotes the differentiation of human B cells into plasma cells and lymphocytes [129].In addition, the antibodies produced by B cells also rely on the decomposition of glutamine. When the expression of ASCT2 and GLS is inhibited, the production of IgG and IgM antibodies will decrease [130].In a tumor microenvironment deficient in glutamine, the function of B cells is inhibited.

Discussion above have presented that the glutamine metabolism also plays a pivotal role in tumor progression. Consequently, the relevant substances in this metabolic pathway also possess the potential to become biomarkers for assessing tumor progression and predicting prognosis. In next part (Part 4), related clinical studies will be discussed.

### Altered lactate metabolic pathways in tumors

Lactate, a glycolytic end-product, accumulates in tumors due to elevated glycolytic flux under the Warburg effect. Lactate regulates metabolism, participates in signaling, and modifies the tumor microenvironment to promote cancer progression [131].

Intracellular lactate levels modulate histone lactylation, thereby altering gene transcription and metabolic regulation. For instance, in NSCLC, lactate reduces the transcription of glycolytic enzymes (HK1 and PKM) while enhancing the expression of TCA cycle enzymes including succinate dehydrogenase complex flavoprotein subunit A (SDHA) and IDH non-catalytic subunit gamma (IDH3G) histone lactylation at gene promoter regions [132]. This process facilitates glucose metabolic reprogramming. Meanwhile, molecules such as SATB2 inhibits growth and metastasis of lung tumor by mediating H3K9 delactylation [133]. Non-histone lactylation also holds significant importance and exerts a more direct effect compared to histone lactylation. It promotes cancer progression by providing energy and facilitating DNA repair, among other pathways [134, 135]. In cancer types such as lung cancer, lactylation of the Nijmegen Breakage Syndrome 1 protein (NBS1) in cancer cells can enhance tumor growth and drug resistance by promoting homologous recombination-mediated DNA repair, thereby reducing patient survival rates [136]. In lung adenocarcinoma (LUAD), lactylation of lactate LDHA in cancer cells promotes glycolysis [137], providing more energy for tumors while generating more lactate to form a positive feedback loop and strengthen other effects of lactate on cancer progression.

In the tumor microenvironment, lactate exerts immunosuppressive effects and promotes metastasis. Treg cells can utilize lactate, while other effector T cells cannot [138]. The high-lactate environment in TME also induces PD-1 expression in Treg cells, whereas PD-1 expression in effector T cells is inhibited [139]. Through multiple mechanisms, lactate suppresses the activation of T cells in TME, thereby impairing anti-tumor immunity. Besides, lactate drives polarization of TAMs toward the M2 phenotype through mechanisms such as promoting histone lysine lactylation to regulate gene expression [140] or directly activating downstream pathways as a signaling molecule [141], which suppresses adaptive immunity and promotes tumor-associated inflammation [142]. Furthermore, lactate stimulates fibroblasts to produce hyaluronic acid, enhancing tumor invasiveness. The resulting extracellular acidosis activates matrix metalloproteinases (MMPs) and cathepsins, promoting extracellular matrix degradation and tumor invasion [143]. Currently, research on the impact of TME lactate on B cells remains limited. However, since B cells express the lactate transporter monocarboxylate transporter 1 (MCT1) on their surface, we hypothesize that the activity of B cells in TME is also affected by lactate.

Overall, lactate is deeply involved in multiple aspects of tumor progression. Accordingly, numerous clinical studies are exploring lactate pathway activity as prognostic indicator; relevant findings will be introduced in next section (Part 4).

### Altered lipid metabolic pathways in tumors

The alterations in lipid metabolism vary among different types of cancers [144]. To meet the demands of membrane biosynthesis, energy production, and growth signaling, most cancer cells upregulate de novo lipogenesis—unlike normal cells, which primarily rely on exogenous fatty acid uptake [145–147]. Some tumors further exploit lipid scavenging from neighboring cancer-associated adipocytes. The fatty acid synthesis pathway initiates with citrate from the TCA cycle, which is sequentially converted by ATP-citrate lyase (ACLY) to acetyl-CoA, by acetyl-CoA carboxylase (ACC) to malonyl-CoA, and finally by fatty acid synthase (FASN) to palmitate. Palmitate is further elongated and desaturated to generate diverse fatty acids (FAs), which are then activated by fatty acid-CoA ligases for storage in lipid droplets (LDs) or utilization in membrane synthesis, protein lipidation, or β-oxidation. To sustain this metabolic reprogramming, cancer cells enhance glutamine consumption and overexpress lipid metabolic enzymes with elevated catalytic activity [148]. Notably, in lung cancer and other malignancies, bidirectional crosstalk between adipocytes and tumor cells establishes a metabolic symbiosis: cancer cells induce adipocyte lipolysis and internalize released FAs via specific transporters (such as clusters of differentiation 36(CD36) and fatty acid transport proteins 1(FATP1)) [149–154]. Clinically, numbers of substances in lipid metabolism pathway correlate with poor prognosis in cancers including lung cancer. For instance, elevated CD36 expression correlates with poor prognosis and metastasis in certain cancers. High expression of the aldehyde dehydrogenase 1 family member l2(ALDH1L2) negatively regulates the level of cellular lipid peroxidation and promoting small cell lung cancer(SCLC) chemoresistance [155] LncRNA *AFAP1-AS1* translated mitochondrial-localized peptide(ATMLP) activates the AKT pathway and promotes LD accumulation, ultimately promoting NSCLC cell survival under ionizing radiation [156] All these studies have underscored the therapeutic relevance of lipid metabolism in oncology.Numerous studies have demonstrated that in lung cancer and other types of malignancies, TME cells other than cancer cells also exhibit alterations in lipid metabolic pathways [157, 158], and these alterations are strongly associated with immunosuppressive effects. This further indicates that lipid metabolites and enzymes involved in related lipid metabolism hold potential as tumor markers for early diagnosis and therapeutic efficacy prediction.

In lung cancer TME, PD-1 signaling inhibits the expression of phospholipid phosphatase 1(PLPP1) via the Akt-GATA1 pathway, leading to insufficient synthesis of phosphatidylcholine(PC) and phosphatidylethanolamine(PE) in CD8⁺ T cells [157]. PLPP1 deficiency promotes ferroptosis in CD8⁺ T cells through mechanisms such as elevated reactive oxygen species(ROS). Meanwhile, unsaturated fatty acids in TME can also stimulate ferroptosis in CD8⁺ T cells with PLPP1 deficiency. Many researches on Treg cells have pointed out that these cells not only take up more free fatty acids(FFAs) [159], but also upregulate de novo fatty acid synthesis [160, 161], promoting its immunosuppressive function.

The metabolic reprogramming of B cells in TME requires further investigation. However, studies using mouse models have revealed that cancer cell-derived metabolites (e.g., leukotriene B4) in certain cancer types can induce the expression of peroxisome proliferator-activated receptor α(PPARα) in B cells [158]. PPARα regulates the expression of genes mediating metabolic processes including fatty acid oxidation(FAO). Additionally, it initiates the differentiation of regulatory B cells(Breg cells).

Abnormal level of the lipid metabolite 25-hydroxycholesterol (25HC) exists in TME of various cancers, including lung squamous cell carcinoma(LUSC) [162]. In TAMs, IL-4 and IL-13 can induce the expression of cholesterol-25-hydroxylase (Ch25h) via the transcription factor STAT6, resulting in the accumulation of 25HC. 25HC accumulated in lysosomes competes with cholesterol for binding to the lysosomal cholesterol signaling protein GPR155, inhibiting the kinase mTORC1 and thereby triggering AMPKα activation and metabolic reprogramming. AMPKα can also enhance the activation of STAT6 and the production of ARG1. Through metabolic regulation, ARG1 inhibits T cell activation, reinforces the immunosuppressive phenotype of TAMs, and promotes tumor growth. Researchers have found that high CH25H expression is associated with poor survival in patients with LUSC and other types of cancers. Numerous studies have shown that lipid anabolism and accumulation in TAMs regulates their polarization, which plays a crucial role in tumor progression and metastasis [163, 164].

Given the broad influence of lipid metabolic reprogramming on both cancer cells and other cells within TME, substances associated with this pathway offer significant potential as important biomarkers for prognostic prediction. We will introduce relevant clinical studies in the following part.

## Clinical application

Since metabolic reprogramming in cancer cells leads to alterations in metabolite levels, we propose that the impacts of treatment on the levels of four metabolites—glucose, lactate, glutamine, and lipids—can be used to evaluate and predict therapeutic efficacy.

For instance, the study by Liu et al. demonstrated that inhibition of the TCA cycle improves anti-PD-1 efficacy in melanoma through ATF3-mediated regulation of PD-L1 and glycolysis. This work exemplifies how targeting glucose metabolism can enhance immunotherapy responses, with measurable metabolic changes serving as pharmacodynamic biomarkers. The study by Luis E. Raez et al. demonstrated that by combining 2-deoxyd-glucose (2DG), which inhibits glycolysis, with docetaxel, the efficacy of docetaxel in non-small cell lung cancer and thyroid cancer can be enhanced. This study demonstrates how targeting glucose metabolism can enhance the efficacy of chemotherapy [165]. However, the sample size of this study is relatively small, and larger-scale cohort validation remains to be carried out.

Another example is the combined application of glutaminase inhibitors telaglenastat and everolimus in the treatment of advanced renal cell carcinoma. In this clinical trial conducted by Lee et al., the experimental group telaglenastat plus everolimus (TelaE) showed a longer progression-free survival (PFS) compared with the control group placebo plus everolimus (PboE) [166](NCT03163667). This indicates that targeting glutamine metabolism can enhance the effectiveness of chemotherapy.

Gerald Falchook et al. conducted a study, in which patients with advanced metastatic solid tumors, including KRAS^MUT^ NSCLC, ovarian cancer, and breast cancer, received oral administration of FASN inhibitor TVB-2640 to inhibit FASN-mediated fatty acid synthesis. It turned out that TVB-2640 effectively enhanced the efficacy of paclitaxel in patients, with predictable and manageable safety profile. In this study, the levels of FASN metabolites (or their surrogates) in serum were used to assess the engagement of the metabolic pathway, and elevated FASN levels before treatment are associated with more pronounced therapeutic efficacy. This cohort study once again effectively confirms that the response of metabolic pathways to treatment is closely related to therapeutic efficacy, and metabolite levels hold promising prospects for predicting efficacy [167](NCT00023247).

Combining mechanistic studies with cohort validation, Shang et al. found that elevated levels of serum lactate and LDH in smalll cell lung cancer(SCLC) patients were associated with less benefits from immunotherapy. This finding not only demonstrates that targeting lactate metabolism can enhance the efficacy of immunotherapy, but also supports our view that lactate levels can be used to predict therapeutic efficacy. However, this study did not further explore specific protocols for the combination of lactate metabolism inhibitors and immunotherapy [168].

Below, we have searched for a series of clinical trials that use these metabolites as indicators to predict therapeutic efficacy or prognosis (Table 4). At the same time, we have summarized the examples of early tumor screening using metabolite profiling in recent years (Table 5). Currently, there is a lack of consensus guidelines or authoritative recommendations specifically endorsing metabolomics-based biomarkers for applications in lung cancer. Existing expert opinions and consensus statements mainly emphasize the potential and research prospects of metabolomics in this field, while highlighting the urgent need for further validation through large-scale, multicenter clinical studies.

**Table 4:** Clinical trials on the association between metabolites and prognosis

Relevant Experiment/Article/Trail Number	Metabolic Pathway	Experimental Protocol	Pathway Changes	Metabolite Type	Metabolite Level	Prognosis/Therapeutic Effect
PMC9198346	Lipid	High-resolution MS analysis of surgical resection tissue samples of NSCLC obtained after neoadjuvant chemotherapy (NAC). [74]	Glycerophospholipid metabolism	Sphingomyelin (SM)	High	Good
				Lysophosphatidylinositol (LysoPI)	Low	Good
				Phosphatidylethanolamine (PE)	Low	Good
				Lysophosphatidylcholine (15:0/0:0)/Lysophosphatidylethanolamine (18:0/0:0)	High	Good
NCT01835041	Glucose and glutamine	Measurement of plasma metabolite levels in melanoma patients by NMR spectroscopy [169]	Increased PDHA1 expression, enhanced TCA cycle	Pyruvate	High	Bad
				Fumarate	High	Bad
				2-Oxoglutarate	High	Bad
NCT03504423	Glucose and glutamine	Folfirinox (FFX) combined with CPI-613 and modified Folfirinox (mFFX) for the treatment of pancreatic metastatic adenocarcinoma	Low expression of mitochondrial pyruvate carrier MPC1 and MPC2 [76, 169]	Pyruvate	High	Bad
NCT03089606	Tryptophan (Trp)	Subjects underwent C11-AMT PET, FDG PET, baseline study tumor biopsy, and received at least one infusion of pembrolizumab as part of the study	Disordered Trp metabolism [77]	Tryptophan	High	Bad
GSE115978, GSE123813	Lactate	Collection and review of publicly available single-cell RNA sequencing (scRNA-seq) cohorts obtained from patients receiving immunotherapy to elucidate the association between liver metastasis (LM) and immunotherapy response	Disordered lactate metabolism [79, 170]	Lactate	High	Bad
PMC11800462	Lactate, Leucine	The study included 33 healthy controls (HC), 38 newly diagnosed multiple myeloma patients (NDMM) divided into three RISS grades, and 92 multiple myeloma patients treated with bortezomib regimen after targeted therapy	Upregulated CKS2 expression, downregulated LYZ expression [80]	Lactate	Low	Bad
				Leucine	Low	Bad

**Table 5:** Early diagnosis using alterations in plasma metabolite levels

Cancer	Key Metabolites	Trend of change	Reference
Lung cancer	Fumaric acid, phosphatidylcholine (LysoPCS) such as LysoPC 20:3 and LysoPC 18:2, β -hydroxybutyric acid, carnitine, N-acetylcysteine, glutamic acid	Rise	[171, 172]
	Phosphatidylcholine such as PC ae C40:6, PC aa C38:0 and PC aa C40:2, tryptophan, sulfur ammonium methane, citrate, 1, 3 (S) -HODE, arachidonic acid, glutamine, threonine, histidine	Decline	[171–173]
Gastric cancer	SAM, neopterin, GlcNAc6p, serotonin, and NMN	Rise	[174]
	Alpha-linolenic acid, linoleic acid, palmitic acid, succinate, uridine, lactate, pyroglutamate, and 2-aminooctanoate	Decline	[174, 175]
Colorectal adenocarcinoma	Kynurenine, dihydrothymosine, oleic acid	Rise	[176]
	Isopropanolic acid, L-histidine and dehydroepiandrosterone (DHEA) sulfates	Decline	
Breast cancer	Ethyl (R) −3-hydroxyhexanoate, octanoic acid	Rise	[177]
Liver cancer	L-glutamic acid, alpha-fetoprotein	Rise	[178]
Pancreatic cancer	β -phytosterols, spengarin	Rise	[179]
	Creatine, inosine	Decline	
Ovarian cancer	Asparagine, methionine, glutamine	Rise	[180]
	Glycolic acid, glutamic acid	Decline	

## Discussion

This review summarizes the strengths and limitations of commonly used metabolite detection methods, highlights the advantages of liquid biopsy in cancer-related applications, and discusses the abnormal features of four key metabolic pathways—glucose, lactate, lipids, and glutamine—in tumors and their associations with cancer prognosis. We highlight metabolite-based biomarkers as promising tools for prognostic prediction and therapeutic monitoring. However, as discussed in 2.2, liquid biopsy for metabolites is largely limited by the instability of metabolites. Therefore, to obtain relatively accurate and reproducible results and enable successful clinical translation, establishing standardized operating procedures(SOPs) for sample collection, preservation and processing is critically important. In fact, some early studies have already standardized sample handling and published their detailed operational protocols [12, 181] Currently, the widely accepted plasma sample processing protocols for metabolomics include fasting for at least 8 h before blood collection, using ethylene diamine tetraacetic acid(EDTA) as the anticoagulant, initiating sample processing within 30 min after collection, transferring plasma into cryotubes, storing samples at −80℃, and thawing them on ice before analysis(NCT06344546,NCT03014856,NCT04287712) [182].Ongoing trials such as NCT06930807 is currently investigating metabolites as a preliminary prognostic biomarker tool, due to their low plasma requirements, cost-effectiveness, and repeatability, demonstrating growing clinical interest.

This review's strengths lie in its focus on major diseases that continue to afflict humanity, particularly cancer. It summarizes common alterations in metabolic pathways associated with cancer, along with variations in corresponding metabolite types and levels, thereby providing a comprehensive, well-organized overview of current research achievements in this field. Building on this foundation, the review begins by examining widely used liquid biopsy techniques, summarizing the most prevalent and cutting-edge technologies and their characteristics. It particularly emphasizes the potential applications of these technologies in predicting tumor prognosis. The review aims to synthesize current findings on the use of metabolites for prognostic predictions and to offer feasible ideas for future studies in this area. However, we acknowledge that this review has certain limitations. It primarily focuses on changes in four metabolic pathways—glucose, glutamine, lipids, and lactate—that significantly influence cell growth and proliferation, while other metabolic pathway alterations receive less attention. Additionally, the scarcity of clinical studies employing metabolites to predict patients' responses to specific treatments or disease prognosis contributes to a lack of data in this review.Despite these limitations, we believe that this review provides a unique perspective and valuable insights that can guide future research endeavors.

## Data Availability

Not applicable.
